# Local and Overall Deviance R-Squared Measures for Mixtures of Generalized Linear Models

**DOI:** 10.1007/s00357-023-09432-4

**Published:** 2023-04-04

**Authors:** Roberto Di Mari, Salvatore Ingrassia, Antonio Punzo

**Affiliations:** Dipartimento di Economia e Impresa, Università di Catania, Catania, Italy

**Keywords:** Generalized linear models, Deviance, R-squared, Maximum likelihood, Mixture models

## Abstract

In generalized linear models (GLMs), measures of lack of fit are typically defined as the deviance between two nested models, and a deviance-based *R*^2^ is commonly used to evaluate the fit. In this paper, we extend deviance measures to mixtures of GLMs, whose parameters are estimated by maximum likelihood (ML) via the EM algorithm. Such measures are defined both locally, i.e., at cluster-level, and globally, i.e., with reference to the whole sample. At the cluster-level, we propose a normalized two-term decomposition of the local deviance into explained, and unexplained local deviances. At the sample-level, we introduce an additive normalized decomposition of the total deviance into three terms, where each evaluates a different aspect of the fitted model: (1) the cluster separation on the dependent variable, (2) the proportion of the total deviance explained by the fitted model, and (3) the proportion of the total deviance which remains unexplained. We use both local and global decompositions to define, respectively, local and overall deviance *R*^2^ measures for mixtures of GLMs, which we illustrate—for Gaussian, Poisson and binomial responses—by means of a simulation study. The proposed fit measures are then used to assess, and interpret clusters of COVID-19 spread in Italy in two time points.

## Introduction

In the framework of the classical linear model, the coefficient of determination, also known as R-squared (*R*^2^), is a widely used goodness of fit measure, whose advantages and limitations are well-known in literature (see, e.g., Cameron and Windmeijer (1997) and Cohen et al. (2013)). For generalized linear models (GLMs), measures of lack of fit are typically defined based on the deviance, which compares the log-likelihoods of two nested GLMs. In this context, the deviance-based *R*^2^ is extensively used to assess goodness of fit. In this paper, we focus on mixtures of GLMs (see, e.g., Wedel and Kamakura, 2000, Chapter 7 and Frühwirth-Schnatter, 2006 Chapter 8), whose parameters are estimated by ML. These models represent a classical generalization of a single GLM, designed to handle data that are clustered around (generalized) linear structures. We introduce different deviance measures considering both cluster, and sample levels.

At cluster-level, where the clusters are identified by the a posteriori soft partition provided by the fitted model (defined as “soft” since each unit’s memberships to the clusters are in the form of probabilities), we define a local deviance. The latter compares, for each cluster, the intercept-only GLM with the saturated GLM. We show that the newly defined local deviance can be decomposed into an explained local deviance, and a remained part, which is left unexplained by the local GLM.

At sample-level, we first define the total deviance by comparing the intercept-only GLM mixture with the saturated GLM mixture. Subsequently, we propose to decompose it into a normalized summation of three terms. Each additive term evaluates a different aspect of the fitted model: cluster separation on the dependent variable, the proportion of total deviance which is explained by the fitted model, the proportion of the total deviance which remains unexplained. We leverage on these new decompositions to define a local, and an overall deviance *R*^2^ measure.

The paper is organized as follows. In Section 2, we set up the baseline framework of GLMs, devoting special emphasis to the concepts of deviance and *R*^2^. In Section 3, we introduce mixtures of GLMs and propose our local and overall deviance measures for this class of models. In Section 4, we derive the normalized decompositions of the local and total deviances, and conclude the section by introducing our local and overall deviance *R*^2^s. The proposal is first illustrated by means of three simulation studies in Section 6—for conditional Gaussian, Poisson, and binomial response types, respectively. The simulation for the Gaussian case includes an evaluation of the impact of choosing among the most popular initialization strategies. In Section 7, our fit measures are used to assess, and interpret clusters of COVID-19 prevalence in Italy in two points in time. We conclude the paper (Section 8) with a final discussion, and possible venues for future work.

## Preliminaries About Generalized Linear Models

Let $(\boldsymbol {x}_{i}^{\prime },y_{i})^{\prime }$, *i* = 1,…,*n*, be independent observations from $(\boldsymbol {X}^{\prime },Y)'$, where *Y* is the dependent (or response) variable and ***X*** is a vector of *d* explanatory variables—which we call covariates, regressors, or predictors interchangeably. In GLMs, we assume that the conditional distribution of *Y*_*i*_ given ***X***_*i*_ = ***x***_*i*_ is a member of the exponential family with probability density (or mass) function

1 $$ f(y_{i};\theta_{i},\phi)=\exp\left\{\frac{y_{i}\theta_{i}-b(\theta_{i})}{a(\phi)}+c(y_{i};\phi)\right\}, $$

where *𝜃*_*i*_ = *𝜃*(***x***_*i*_) is the canonical parameter, which is a function of ***x***_*i*_, *ϕ* (if unknown) is a dispersion parameter, and *a*(⋅), *b*(⋅), and *c*(⋅) are known functions varying from one member of the family to another. If *ϕ* is known, the model (1) is a member of the (one-parameter) exponential family; if *ϕ* is unknown, the model (1) may, or may not be a member of the two-parameter exponential family. The function *b*(⋅) describes the relationship between the mean of *Y*_*i*_, denoted by *μ*_*i*_, and the canonical parameter *𝜃*_*i*_, given by $ \mu _{i}=b^{\prime }(\theta _{i}) $, where prime denotes differentiation with respect to *𝜃*_*i*_ (see, e.g., McCullagh & Nelder, 1989, pp. 28–29).

In GLMs, a monotone and differentiable link function $g\left (\cdot \right )$ is exploited to relate *μ*_*i*_ to the covariates ***x***_*i*_ through the relation

2 $$ g(\mu_{i})=g[\mu(\boldsymbol{x}_{i};\boldsymbol{\beta})]=\eta(\boldsymbol{x}_{i};\boldsymbol{\beta})=\eta_{i}, $$

where $\eta (\boldsymbol {x}_{i};\boldsymbol {\beta })=\boldsymbol {\beta }^{\prime }\boldsymbol {x}_{i}^{*}$ is the so-called linear predictor, with $\boldsymbol {\beta } \in \mathbb {R}^{d+1}$ and $\boldsymbol {x}_{i}^{*}=(1,\boldsymbol {x}_{i}^{\prime })'$ to include the intercept term. Note that (2) can be inverted to obtain *μ*_*i*_ = *g*^− 1^(*η*_*i*_). As far as the choice of *g*(⋅) is concerned, more specialized results can be obtained by choosing the canonical link function such that *𝜃*_*i*_ = *η*_*i*_, and the density in (1) can be easily expressed as a function of *μ*_*i*_, rather than using the canonical parameter *𝜃*_*i*_, as

3 $$ f(y_{i};\mu_{i},\phi)=\exp\left\{\frac{y_{i} g^{-1}\left( \mu_{i}\right)-b[g^{-1}\left( \mu_{i}\right)]}{a(\phi)}+c(y_{i};\phi)\right\}. $$

Table 1 specifies all the quantities defined so far for three well-known members of the exponential family: the Gaussian, Poisson, and binomial distributions.

**Table 1:** Characteristics of some common distributions in the exponential family; refer to models (1)–(3)

		Gaussian	Poisson	Binomial
Exponential-family		Two-parameter	One-parameter	One-parameter
Notation		$\mathcal {N}(\mu _{i},\sigma ^{2})$	$\mathcal {P}(\mu _{i})$	${\mathscr{B}}(m,p_{i})/m$
Support of *Y*_*i*_		$(-\infty ,\infty )$	$\left \{0,1,\ldots \right \}$	$\left \{0/m,1/m,\ldots ,m/m\right \}$
Canonical link *g*(⋅)		Identity	Log	Logit
*a*(*ϕ*)		*σ*^2^	1	1/*m*
*b*(*𝜃*_*i*_)		${\theta _{i}^{2}}/2$	$\exp (\theta _{i})$	$\ln \left [1+\exp (\theta _{i})\right ]$
*c*(*y*_*i*_; *ϕ*)		$-\displaystyle \frac {1}{2}\left [\frac {{y_{i}^{2}}}{\phi }+\ln (2\pi \phi )\right ]$	$-\ln (y_{i}!)$	$\ln \displaystyle \binom {m}{my_{i}}$
*μ*(*𝜃*_*i*_)		*𝜃*_*i*_	$\exp (\theta _{i})$	$\displaystyle \frac {\exp (\theta _{i})}{1+\exp (\theta _{i})}$
Var(*Y*_*i*_)		*σ*^2^	*μ*_*i*_	*p*_*i*_(1 − *p*_*i*_)/*m*

### Maximum Likelihood Estimation

For GLMs, the estimates of the regression coefficients ***β***, and of the dispersion parameter *ϕ* (in the case of a two-parameter exponential family distribution), are typically obtained by the ML method. The log-likelihood function for a GLM, with density in Eq. 3, is given by

4 $$ \ell\left( \boldsymbol{\mu},\phi\right) = \sum\limits_{i=1}^{n} \ln\left[f(y_{i};\mu_{i},\phi)\right] = \frac{1}{a(\phi)}\sum\limits_{i=1}^{n} \left\{y_{i} g^{-1}(\mu_{i})-b\left[g^{-1}(\mu_{i})\right]\right\} + \sum\limits_{i=1}^{n} c(y_{i};\phi), $$

where ***μ*** is an *n* × 1 vector, with *i* th entry equal to *μ*_*i*_. We denote by $\widehat {\boldsymbol {\beta }}$, and $\widehat {\phi }$ the ML estimates of ***β*** and *ϕ*, respectively, and by $\widehat {\mu }_{i}=\mu \left (\boldsymbol {x}_{i};\widehat {\boldsymbol {\beta }}\right )$ the fitted value of *Y*_*i*_.

### Lack of Fit

In GLMs, the deviances replace the sums of squares (SS) of ordinary least squares (OLS) regression as the building blocks to define measures of lack of fit to the data of the GLM (see, e.g., Cohen et al., 2013). Notably, deviance measures are built from the maximum log-likelihoods of three models: the most parsimonious intercept-only model (*null model*), the model we are interested in (*fitted model*), and the least parsimonious model, with *n* parameters, providing a perfect fit (*saturated model*). The null and saturated models are defined so that $\widehat {\mu }_{i}=\bar {y}$, and $\widehat {\mu }_{i}=y_{i}$ (*i* = 1,…,*n*), respectively, with $\bar {y}$ being the sample mean of *Y*.

Each deviance is a measure of lack of fit, and is obtained as twice the difference between the log-likelihood of one model, compared to the log-likelihood of another (nested) model. Therefore, the larger the value of deviance for the nested model, the worse its goodness of fit. The two most used deviances are the null deviance

5 $$ D\left( \bar{\boldsymbol{y}},\boldsymbol{y};\widehat{\phi}\right)=2\left[\ell\left( \boldsymbol{y},\widehat{\phi}\right)-\ell\left( \bar{\boldsymbol{y}},\widehat{\phi}\right)\right] $$

and the fitted deviance

6 $$ D\left( \widehat{\boldsymbol{\mu}},\boldsymbol{y};\widehat{\phi}\right)=2\left[\ell\left( \boldsymbol{y},\widehat{\phi}\right)-\ell\left( \widehat{\boldsymbol{\mu}},\widehat{\phi}\right)\right], $$

where ***y***, $\widehat {\boldsymbol {\mu }}$, and $\bar {\boldsymbol {y}}$ are the *n* × 1 vectors with *i* th entry being *y*_*i*_, $\widehat {\mu }_{i}$, and $\bar {y}$, respectively, and $\widehat {\phi }$ is the ML estimate of *ϕ* under the fitted model. As well-motivated by Cameron and Windmeijer (1996), the same estimate of *ϕ* is used for all the models (null, fitted and saturated), as we wish to measure the fit due to the regressors, and not to the dispersion parameter.

The null deviance in Eq. 5 is analogous to the total sum of squares (TSS), that is, the total variation in the dependent variable *Y* from the OLS regression. This measures the discrepancy between the worst, and the best possible models, i.e., all the discrepancy that the (fitted) model can potentially account for.

The fitted deviance in Eq. 6 is analogous to the residual sum of squares (RSS) from OLS regression. This deviance measures the lack of fit after modeling with *d* predictors. Even if it is not as widespread in the literature, in principle we could also define a sort of “explained” deviance as

7 $$ D\left( \bar{\boldsymbol{y}},\widehat{\boldsymbol{\mu}};\widehat{\phi}\right)=2\left[\ell\left( \widehat{\boldsymbol{\mu}},\widehat{\phi}\right)-\ell\left( \bar{\boldsymbol{y}},\widehat{\phi}\right)\right], $$

which compares the null and fitted models, analogously to the explained sum of squares (ESS) from OLS regression.

Explained and residual deviances allow us to decompose the null deviance as

8 $$ D\left( \bar{\boldsymbol{y}},\boldsymbol{y};\widehat{\phi}\right) = D\left( \bar{\boldsymbol{y}},\widehat{\boldsymbol{\mu}};\widehat{\phi}\right) + D\left( \widehat{\boldsymbol{\mu}},\boldsymbol{y};\widehat{\phi}\right). $$

### Deviance *R*^2^

In OLS regression, the *R*^2^ is a universal and agreed-upon index of model goodness of fit measuring the proportion of total variation in *Y* accounted for by a set of *d* predictors. No single agreed-upon index of goodness of fit exists for GLMs, although several approaches have been proposed. These pseudo-*R*^2^s are based on different definitions of residuals, the most common of which are the raw residuals, the Pearson residuals, and the deviance residuals (Cameron & Windmeijer, 1996). Note that none of these indexes is a goodness of fit measure, in the sense that none can be interpreted as “proportion of variance accounted for,” as in the OLS regression.

For GLMs, the deviance *R*^2^ is one of the favorite indexes of goodness of fit by applied and theoretical researchers (Cameron & Windmeijer, 1997 and Menard, 2002); it is based on the deviance residuals, and it is sometimes the only measure discussed in articles/textbooks (Guisan & Zimmermann, 2000) and implemented in statistical software (Crawley, 2012, Chapter 13). Intuitively, it looks similar to the *R*^2^ = ESS/TSS of simple linear regression, where the sums of squares are replaced with the deviance measures defined in (5) and (7). Its formula is given by

9 $$ \begin{array}{@{}rcl@{}} R^{2} &=& 1 - \frac{D\left( \widehat{\boldsymbol{\mu}},\boldsymbol{y};\widehat{\phi}\right)}{D\left( \bar{\boldsymbol{y}},\boldsymbol{y};\widehat{\phi}\right)} \end{array} $$

10 $$ \begin{array}{@{}rcl@{}} &=& \frac{D\left( \bar{\boldsymbol{y}},\widehat{\boldsymbol{\mu}};\widehat{\phi}\right)}{D\left( \bar{\boldsymbol{y}},\boldsymbol{y};\widehat{\phi}\right)} . \end{array} $$

Due to the two-term decomposition (8), also this index ranges between zero and one.

## Deviance Decompositions for Mixtures of GLMs

### Mixtures of GLMs and Their Complete-Data Log-Likelihood

Suppose that the conditional expectation of *Y*_*i*_ given ***X***_*i*_ = ***x***_*i*_ varies across the *k* levels (groups, clusters or classes), labeled as 1,…,*k*, of a categorical (nominal) latent variable *J*_*i*_. Under this assumption, mixtures of GLMs constitute a reference framework of analysis (see, e.g., (McLachlan & Peel, 2000, Chapter 5) and (Wedel & Kamakura, 2000, Chapter 7)).

The density of a generic observation *y*_*i*_, according to a mixture of *k* GLMs, can be written as

11 $$ f(y_{i};\boldsymbol{\psi}) = \sum\limits_{j=1}^{k} \pi_{j} f(y_{i};\mu_{ij},\phi_{j}),  $$

where *f*(*y*_*i*_;*μ*_*ij*_,*ϕ*_*j*_) is the *j* th component density of *y*_*i*_ defined like in Eq. 3, with mean $\mu _{ij}=\text {E}\left (Y_{i}|\boldsymbol {X}_{i}=\boldsymbol {x}_{i},J_{i}=j\right )$ and dispersion parameter *ϕ*_*j*_ (in the case of a two-parameter exponential family), and *π*_*j*_ = *P*(*J*_*i*_ = *j*), with *π*_*j*_ > 0 and ${\sum }_{j=1}^{k} \pi _{j}=1$. In Eq. 11, ***ψ*** denotes the set of all parameters of the model, where *π*_1_,…,*π*_*k*− 1_ are the weights, ***β***_1_,…,***β***_*k*_ are the local regression coefficients, and, in the case of a two-parameter exponential family distribution for *Y*_*i*_|***X***_*i*_ = ***x***_*i*_,*J*_*i*_ = *j*, *ϕ*_1_,…,*ϕ*_*k*_ are the local dispersion parameters. General conditions for the identifiability of model Eq. 11 are given in Grün and Leisch (2008a) (see also Frühwirth-Schnatter (2006)).

ML estimates of the parameters are usually obtained via the expectation-maximization (EM) algorithm (Dempster et al., 1977). The core quantity of the algorithm is the complete-data log-likelihood

12 $$ \ell_{c}\left( \boldsymbol{\psi}\right) = \sum\limits_{j=1}^{k}\sum\limits_{i=1}^{n} z_{ij} \ln \pi_{j} + \sum\limits_{j=1}^{k} \ell_{\text{GLM},j}\left( \boldsymbol{\mu}_{j},\phi_{j}\right) , $$

where, based on Eq. 4,

13 $$ \begin{array}{@{}rcl@{}} \ell_{\text{GLM},j}\left( \boldsymbol{\mu}_{j},\phi_{j}\right) & = & \sum\limits_{i=1}^{n} z_{ij} \ln\left[f(y_{i};\mu_{ij},\phi_{j})\right] \\ & = & \frac{1}{a(\phi_{j})}\sum\limits_{i=1}^{n} z_{ij} \left\{y_{i} g^{-1}(\mu_{ij}) - b\left[g^{-1}(\mu_{ij})\right]\right\} + \sum\limits_{i=1}^{n} z_{ij} c(y_{i};\phi_{j}), \end{array} $$

with *z*_*ij*_ = 1 if $(\boldsymbol {x}_{i}^{\prime },y_{i})'$ comes from the *j* th component, and *z*_*ij*_ = 0 otherwise.

The EM algorithm iterates between the expectation-step (E-step) and the maximization-step (M-step) until convergence. At the generic iteration, in the E-step, given the current estimate of ***ψ*** from the previous iteration, say $\dot {\boldsymbol {\psi }}$, each *z*_*ij*_ in Eq. 12 is replaced by the corresponding a posteriori probability of group membership (expectation of *Z*_*ij*_), say $\ddot {z}_{ij}$. This leads to the expected complete-data log-likelihood function. In the M-step, this function is maximized with respect to ***ψ***; as the two terms on the right-hand side of Eq. 12 have zero cross-derivatives, they can be maximized separately.

The maximization of the expected complete-data log-likelihood function is equivalent to the maximization problem discussed in Section 2.1 (for the complete data), except that each observation $(\boldsymbol {x}_{i}^{\prime },y_{i})'$ contributes to the log-likelihood with a known weight $\ddot {z}_{ij}$ (Ingrassia et al., 2015, Punzo and Ingrassia, 2015, and Mazza et al., 2018). See, e.g., (Wedel & De Sarbo, 1995), and (Wedel & Kamakura, 2000, pp. 120–124) for a complete review of the EM algorithm for fitting the model Eq. 11.

Once the model Eq. 11 is fitted, each observation $(\boldsymbol {x}_{i}^{\prime },y_{i})'$ is classified into one of the *k* categories according to the maximum a posteriori probability (MAP) estimate: $\text {MAP}(\widehat {z}_{ij})=1$ if $ \max \limits _{h}\{\widehat {z}_{ih}\}$ occurs in cluster *j* (*j* = 1,…,*k*), and 0 otherwise, where $\widehat {z}_{ij}$ denotes the output value of $\ddot {z}_{ij}$ at convergence of the EM algorithm.

### Deviance Measures

Based on the arguments given earlier, hereafter we introduce the deviance measures of lack of fit for the mixture model Eq. 11 both locally (for each group *j* defined by the soft partition $\widehat {z}_{ij}$, *i* = 1,…,*n*), and overall (for the whole sample).

Let us introduce what we call the local null model, i.e., the local intercept-only model. In notation terms, such a model assumes $\widehat {\mu }_{ij}=\bar {y}_{j}$, where $\bar {y}_{j}={\sum }_{i=1}^{n}\widehat {z}_{ij}y_{i}/\widehat {n}_{j}$. The quantity $\widehat {n}_{j}={\sum }_{i=1}^{n}\widehat {z}_{ij}$ denotes the expected (soft) size of the *j* th group according to the fitted model, *j* = 1,…,*k*. The local null model can be considered as in between the null and the fitted models. The wording “soft” is used because the group memberships $\widehat {z}_{ij}$, *i* = 1,…,*n* and *j* = 1,…,*k*, are a posteriori probabilities—and not “hard” 0/1 values.

For each soft group *j*, *j* = 1,…,*k*, we define the local null deviance (or simply local deviance)

14 $$ \begin{array}{@{}rcl@{}} \text{D}_{j} = D_{j}\left( \bar{\boldsymbol{y}}_{j},\boldsymbol{y};\widehat{\phi}_{j}\right) & =& 2\left[\ell_{\text{GLM},j}\left( \boldsymbol{y},\widehat{\phi}_{j}\right) - \ell_{\text{GLM},j}\left( \bar{\boldsymbol{y}}_{j},\widehat{\phi}_{j}\right)\right] \\ & =& \frac{2}{a\left( \widehat{\phi}_{j}\right)}\sum\limits_{i=1}^{n} \widehat{z}_{ij} \left[y_{i}\left( y_{i} - \bar{y}_{j}\right) - b\left( y_{i}\right) + b\left( \bar{y}_{j}\right)\right], \end{array} $$

the local fitted deviance (or local residual deviance)

15 $$ \begin{array}{@{}rcl@{}} \text{RD}_{j} = D_{j}\left( \widehat{\boldsymbol{\mu}}_{j},\boldsymbol{y};\widehat{\phi}_{j}\right) & =& 2\left[\ell_{\text{GLM},j}\left( \boldsymbol{y},\widehat{\phi}_{j}\right)-\ell_{\text{GLM},j}\left( \widehat{\boldsymbol{\mu}}_{j},\widehat{\phi}_{j}\right)\right] \\ & =& \frac{2}{a\left( \widehat{\phi}_{j}\right)}\sum\limits_{i=1}^{n} \widehat{z}_{ij} \left[y_{i}\left( y_{i} - \widehat{\mu}_{ij}\right) - b\left( y_{i}\right) + b\left( \widehat{\mu}_{ij}\right)\right], \end{array} $$

and the local explained deviance

16 $$ \begin{array}{@{}rcl@{}} \text{ED}_{j} = D_{j}\left( \bar{\boldsymbol{y}}_{j},\widehat{\boldsymbol{\mu}}_{j};\widehat{\phi}_{j}\right) & =& 2\left[\ell_{\text{GLM},j}\left( \widehat{\boldsymbol{\mu}}_{j},\widehat{\phi}_{j}\right)-\ell_{\text{GLM},j}\left( \bar{\boldsymbol{y}}_{j},\widehat{\phi}_{j}\right)\right] \\ & =& \frac{2}{a\left( \widehat{\phi}_{j}\right)}\sum\limits_{i=1}^{n} \widehat{z}_{ij} \left[y_{i}\left( \widehat{\mu}_{ij} - \bar{y}_{j}\right) - b\left( \widehat{\mu}_{ij}\right) + b\left( \bar{y}_{j}\right)\right], \end{array} $$

where $\widehat {\boldsymbol {\mu }}_{j}$ and $\bar {\boldsymbol {y}}_{j}$ are *n* × 1 vectors with *i* th entry being $\widehat {\mu }_{ij}$, and $\bar {y}_{j}$, respectively.

In (14)–(16), since the focus is on measuring the fit due to the regressors, all considered models (null, fitted, and saturated) are evaluated at the soft partition $\widehat {z}_{ij}$ (*i* = 1,…,*n* and *j* = 1,…,*k*), and at the ML estimate $\widehat {\phi }_{j}$ of *ϕ*_*j*_ under the fitted model (refer to Section 2.2 and to (Cameron & Windmeijer, 1996)). As a consequence, the estimate of the weight *π*_*j*_ is the same for all models, and this is the reason why it vanishes (by simplification) from Eqs. 14–16. In analogy with Eq. 8, it is easy to realize that

17 $$ \text{D}_{j} = \text{ED}_{j} + \text{RD}_{j}, $$

for *j* = 1,…,*k*.

For the full sample, we define the null deviance (or total deviance)

18 $$ \begin{array}{@{}rcl@{}} \text{TD} = D\left( \bar{\boldsymbol{y}},\boldsymbol{y};\widehat{\boldsymbol{\phi}}\right) & = 2\sum\limits_{j=1}^{k}\left[\ell_{\text{GLM},j}\left( \boldsymbol{y},\widehat{\phi}_{j}\right)-\ell_{\text{GLM},j}\left( \bar{\boldsymbol{y}},\widehat{\phi}_{j}\right)\right] \\ & = 2\sum\limits_{j=1}^{k}\frac{1}{a\left( \widehat{\phi}_{j}\right)}\sum\limits_{i=1}^{n} \widehat{z}_{ij} \left[y_{i}\left( y_{i} - \bar{y}\right) - b\left( y_{i}\right) + b\left( \bar{y}\right)\right], \end{array} $$

the (soft) within-group deviance (or simply within deviance)

19 $$ \text{WD} = \sum\limits_{j=1}^{k} \text{D}_{j},  $$

and the (soft) between-group deviance (or simply between deviance)

20 $$ \text{BD} = \sum\limits_{j=1}^{k} \text{BD}_{j},  $$

where

21 $$ \begin{array}{@{}rcl@{}} \text{BD}_{j} = D_{j}\left( \bar{\boldsymbol{y}},\bar{\boldsymbol{y}}_{j};\widehat{\boldsymbol{\phi}}\right) & = & 2\sum\limits_{j=1}^{k}\left[\ell_{\text{GLM},j}\left( \bar{\boldsymbol{y}}_{j},\widehat{\phi}_{j}\right)-\ell_{\text{GLM},j}\left( \bar{\boldsymbol{y}},\widehat{\phi}_{j}\right)\right] \\ & =& 2\sum\limits_{j=1}^{k}\frac{1}{a\left( \widehat{\phi}_{j}\right)}\sum\limits_{i=1}^{n} \widehat{z}_{ij} \left[y_{i}\left( \bar{y}_{j} - \bar{y}\right) - b\left( \bar{y}_{j}\right) + b\left( \bar{y}\right)\right]. \end{array} $$

The between deviance in Eq. 20 measures the discrepancy between the intercept-only model, and the local intercept-only model. In terms of clustering validation:

1. BD can be seen as a separation measure (Cerdeira et al., 2012) see, e.g., indicating how well-separated clusters (represented by $\bar {y}_{1},\ldots ,\bar {y}_{k}$) are along the *y*-axis (the greater the value of BD, the more “separated” the clusters are along *Y* );
2. WD measures the discrepancy between the best possible saturated model, and the local intercept-only model. WD can be seen as a compactness measure (see, e.g., Panagiotakis, 2015), quantifying how close observations in a cluster are with respect to the average response of that cluster (the smaller the value of WD, the more “compact” the clusters are around their average response).

From Eqs. 19 and 20, we obtain the two-term decomposition of the total deviance as

22 $$ \text{TD} = \text{WD} + \text{BD} . $$

The within deviance WD can be further decomposed as

23 $$ \text{WD} = \text{EWD} + \text{RWD} , $$

where

24 $$ \text{EWD} = \sum\limits_{j=1}^{k} \text{ED}_{j}  $$

is the explained within deviance, and

25 $$ \text{RWD} = \sum\limits_{j=1}^{k} \text{RD}_{j}  $$

is the residual within deviance.

Two important remarks follow.

1. The explained within deviance EWD measures the discrepancy between the fitted model and the local intercept-only model; in particular, based on Eq. 23, EWD can be considered as the part of WD explained by the local models involving the covariates.
2. The residual within deviance RWD measures the discrepancy between the best possible saturated model, and the fitted model; specifically, based on Eq. 23, RWD can be considered as the part of WD which we are not able to predict locally based on the covariates.

Finally, substituting (23) in (22), we obtain the final three-term decomposition of the total deviance as

26 $$ \text{TD} = \text{BD} + \text{EWD} + \text{RWD} .  $$

As a special case, when *k* = 1, the BD term in Eq. 26 vanishes and TD = EWD + RWD, which is the null deviance decomposition Eq. 8 for the GLM.

### Some Special Cases

For illustrative purposes, we compute the local deviance measures, introduced in Section 3.2, in the case of three well-known exponential family distributions for $Y_{i}|\left (\boldsymbol {X}_{i}=\boldsymbol {x}_{i},J_{i}=j\right )$: Gaussian (Section 3.3.1), Poisson (Section 3.3.2), and binomial (Section 3.3.3).

#### Gaussian Case

The Gaussian distribution is the only two-parameter exponential family distribution we consider herein. This choice for the response variable in model Eq. 11 leads to mixtures of linear Gaussian regressions. For these mixtures, we recall that measures of lack of fit, based on sums of squares, have been already introduced in (Ingrassia & Punzo, 2020).

Using the notation in Table 1, we are assuming that $Y_{i}|(\boldsymbol {X}_{i}=\boldsymbol {x}_{i},J_{i}=j) \sim \mathcal {N}\left (\mu _{ij},{\sigma ^{2}_{j}}\right )$. Simple algebra allows to simplify the local deviances in Eqs. 14, 15, 16, and Eq. 21, yielding

$$ \begin{array}{@{}rcl@{}} \text{D}_{j} = D_{j}\left( \bar{\boldsymbol{y}}_{j},\boldsymbol{y};{\widehat{\sigma}^{2}_{j}}\right) & =& \frac{1}{{\widehat{\sigma}^{2}_{j}}} \sum\limits_{i=1}^{n} \widehat{z}_{ij} \left( y_{i} - \bar{y}_{j}\right)^{2}, \\ \text{RD}_{j} = D_{j}\left( \widehat{\boldsymbol{\mu}}_{j},\boldsymbol{y};{\widehat{\sigma}^{2}_{j}}\right) & =& \frac{1}{{\widehat{\sigma}^{2}_{j}}} \sum\limits_{i=1}^{n} \widehat{z}_{ij} \left( y_{i} - \widehat{\mu}_{ij}\right)^{2}, \\ \text{ED}_{j} = D_{j}\left( \bar{\boldsymbol{y}}_{j},\widehat{\boldsymbol{\mu}}_{j};{\widehat{\sigma}^{2}_{j}}\right) & =& \frac{1}{{\widehat{\sigma}^{2}_{j}}} \sum\limits_{i=1}^{n} \widehat{z}_{ij} \left( \widehat{\mu}_{ij} - \bar{y}_{j}\right)^{2}, \\ \text{BD}_{j} = D_{j}\left( \bar{\boldsymbol{y}},\bar{\boldsymbol{y}}_{j};{\widehat{\sigma}^{2}_{j}}\right) & =& \frac{1}{{\widehat{\sigma}^{2}_{j}}} \sum\limits_{i=1}^{n} \widehat{z}_{ij} \left( \bar{y}_{j} - \bar{y}\right)^{2} = \widehat{n}_{j} \frac{\left( \bar{y}_{j} - \bar{y}\right)^{2}}{{\widehat{\sigma}^{2}_{j}}}. \end{array} $$

These deviance-based lack of fit measures differ from those in (Ingrassia & Punzo, 2020) as now the component error variances ${\widehat {\sigma }^{2}_{j}}$ enter the decomposition. In terms of residuals, this means that, while the soft raw residuals are considered in (Ingrassia & Punzo, 2020), here the soft Pearson (standardized) residuals (which in this case coincide with the deviance residuals) are considered. This is a favorable improvement allowing us to compare the fit between clusters with different local conditional variances ${\widehat {\sigma }^{2}_{1}},\ldots ,{\widehat {\sigma }^{2}_{k}}$.

#### Poisson Case

Using the notation in Table 1, here we assume that $Y_{i}|(\boldsymbol {X}_{i}=\boldsymbol {x}_{i},J_{i}=j) \sim \mathcal {P}\left (\mu _{ij}\right )$. Simple algebra leads to simplify the local deviances in Eqs. 14, 15, 16, and 21, so to obtain

$$ \begin{array}{@{}rcl@{}} \text{D}_{j} = D_{j}\left( \bar{\boldsymbol{y}}_{j},\boldsymbol{y}\right) & =& 2 \sum\limits_{i=1}^{n} \widehat{z}_{ij} \left[y_{i}\left( \ln y_{i} - \ln \bar{y}_{j}\right) - y_{i} + \bar{y}_{j}\right], \\ \text{RD}_{j} = D_{j}\left( \widehat{\boldsymbol{\mu}}_{j},\boldsymbol{y}\right) & =& 2 \sum\limits_{i=1}^{n} \widehat{z}_{ij} \left[y_{i}\left( \ln y_{i} - \ln \widehat{\mu}_{ij}\right) - y_{i} + \widehat{\mu}_{ij}\right], \\ \text{ED}_{j} = D_{j}\left( \bar{\boldsymbol{y}}_{j},\widehat{\boldsymbol{\mu}}_{j}\right) & =& 2 \sum\limits_{i=1}^{n} \widehat{z}_{ij} \left[y_{i}\left( \ln \widehat{\mu}_{ij} - \ln \bar{y}_{j}\right) - \widehat{\mu}_{ij} + \bar{y}_{j}\right], \\ \text{BD}_{j} = D_{j}\left( \bar{\boldsymbol{y}},\bar{\boldsymbol{y}}_{j}\right) & =& 2 \sum\limits_{i=1}^{n} \widehat{z}_{ij} \left[y_{i}\left( \ln \bar{y}_{j} - \ln \bar{y}\right) - \bar{y}_{j} + \bar{y}\right] . \end{array} $$

#### Binomial Case

Using the notation in Table 1, here we assume that $Y_{i}|(\boldsymbol {X}_{i}=\boldsymbol {x}_{i},J_{i}=j) \sim {\mathscr{B}}\left (m,p_{ij}=\mu _{ij}/m\right )$. Simple algebra leads to simplify the local deviances in Eq. 14, 15, 16, and 21, yielding

$$ \begin{array}{@{}rcl@{}} \text{D}_{j} = D_{j}\left( \bar{\boldsymbol{y}}_{j},\boldsymbol{y}\right) & =& 2 \sum\limits_{i=1}^{n} \widehat{z}_{ij} \left[y_{i}\ln \frac{y_{i}}{\bar{y}_{j}} + \left( m - y_{i}\right) \ln \frac{m-y_{i}}{m-\bar{y}_{j}}\right], \\ \text{RD}_{j} = D_{j}\left( \widehat{\boldsymbol{\mu}}_{j},\boldsymbol{y}\right) & =& 2 \sum\limits_{i=1}^{n} \widehat{z}_{ij} \left[y_{i}\ln \frac{y_{i}}{\widehat{\mu}_{ij}} + \left( m - y_{i}\right) \ln \frac{m-y_{i}}{m-\widehat{\mu}_{ij}}\right], \end{array} $$

$$ \begin{array}{@{}rcl@{}} \text{ED}_{j} = D_{j}\left( \bar{\boldsymbol{y}}_{j},\widehat{\boldsymbol{\mu}}_{j}\right) & =& 2 \sum\limits_{i=1}^{n} \widehat{z}_{ij} \left[y_{i}\ln \frac{\widehat{\mu}_{ij}}{\bar{y}_{j}} + \left( m - y_{i}\right) \ln \frac{m-\widehat{\mu}_{ij}}{m-\bar{y}_{j}}\right], \\ \text{BD}_{j} = D_{j}\left( \bar{\boldsymbol{y}},\bar{\boldsymbol{y}}_{j}\right) & =& 2 \sum\limits_{i=1}^{n} \widehat{z}_{ij} \left[y_{i}\ln \frac{\bar{y}_{j}}{\bar{y}} + \left( m - y_{i}\right) \ln \frac{m-\bar{y}_{j}}{m-\bar{y}}\right]. \end{array} $$

## Evaluating the Main Aspects of the Fitted Model

### Normalized Three-Term Decomposition of the Total Deviance

Starting from the three-term decomposition of the total deviance in Eq. 26, it is possible to define normalized deviance measures which evaluate the main aspects of the fitted model. In particular, dividing both sides of Eq. 26 by TD, we obtain

27 $$ \begin{array}{@{}rcl@{}} \frac{\text{BD}}{\text{TD}} + \frac{\text{EWD}}{\text{TD}} + \frac{\text{RWD}}{\text{TD}} & = 1 \\ \text{NBD} + \text{NEWD} + \text{NRWD} & = 1, \end{array} $$

where NBD, NEWD, and NRWD are the normalized versions of BD, EWD, and RWD, respectively.

In terms of interpretation, NBD is the proportion of the total deviance explained by the separation measure BD; hence, NBD can seen as a sort of correlation ratio measuring the association between the dependent variable *Y*, and the latent group variable *J*. NEWD is the proportion of the total deviance explained by the inclusion of the covariates ***X***—through the slope(s) of the local regressions. Instead, NRWD represents the proportion of the total deviance, which remains unexplained by the fitted model.

### Normalized Explained Deviance

Exploiting Eq. 27, it is natural to introduce the quantity

28 $$ \text{NED} = \text{NBD} + \text{NEWD} = 1-\text{NRWD}.  $$

NED represents the proportion of the total deviance explained by the fitted model, desirably assuming values in the interval $\left [0,1\right ]$. The larger its value (hence, the smaller NRWD), the better the fit of the mixture of GLMs to the observed data.

Provided that TD > 0, the limit cases NED = 0 and NED = 1 are obtained when NBD = NEWD = 0 and NRWD = 0, respectively. Cases where either of the three terms NBD, NEWD, and NRWD are null, are analyzed below.

- NBD = 0 when BD = 0, which occurs when $\bar {y}_{1}=\cdots =\bar {y}_{k}=\bar {y}$, regardless of the soft group sizes $\widehat {n}_{1},\ldots ,\widehat {n}_{k}$ (see Eq. 21).
- NEWD = 0 when EWD = 0, that is, when $\widehat {\mu }_{ij}=\bar {y}_{j}$, *i* = 1,…,*n* and *j* = 1,…,*k*, regardless of the values of $\widehat {z}_{ij}$ (see Eq. 16).
- NRWD = 0 when RWD = 0. A sufficient condition for the latter equality to be true, regardless of the values of $\widehat {z}_{ij}$, is represented by *k* overlapped component regression lines (i.e., $\widehat {\mu }_{i1}=\cdots =\widehat {\mu }_{ik}=\widehat {\mu }_{i}$, *i* = 1,…,*n*), with all the *n* data points lying on the resulting common regression line (i.e., $y_{i} = \widehat {\mu }_{i}$, *i* = 1,…,*n*) (see Eqs. 15 and 25).

### Local and Overall Deviance *R*^2^ Measures

Leveraging on Eq. 10, it is also natural to define the local deviance *R*^2^ for the *j* th group as

29 $$ {R^{2}_{j}}= \frac{\text{ED}_{j}}{\text{D}_{j}}.  $$

${R^{2}_{j}}$ can be seen as the proportion of the local deviance in the *j* th group that cannot be explained by the intercept-only GLM in that group, but which can by the linear predictor $\eta _{ij}=\widehat {\boldsymbol {\beta }}_{j}^{\prime }\boldsymbol {x}_{i}^{*}$ of the GLM.

As a general note, the higher the ${R^{2}_{j}}$, the better the *j* th GLM fits the data in the *j* th group. In other words, the larger the fraction of local deviance in group *j* that is accounted for by the *j* th GLM, the closer the data points are to the fitted cluster’s regression line.

With the same principle, it is natural to define the overall deviance *R*^2^ as

30 $$ R^{2} = \frac{\text{EWD}}{\text{WD}}.  $$

Intuitively, the overall *R*^2^ in Eq. 30 can be interpreted as the proportion of the within deviance explained (accounted for) by the fitted mixture of GLMs.

Based on Eq. 24, *R*^2^ is related to ${R^{2}_{1}},\ldots ,{R^{2}_{k}}$ by the following relation

31 $$ R^{2} = \frac{\displaystyle\sum\limits_{j=1}^{k} \text{ED}_{j}}{\text{WD}} = \frac{\displaystyle\sum\limits_{j=1}^{k} \text{D}_{j} \frac{\text{ED}_{j}}{\text{D}_{j}}}{\text{WD}} = \frac{\displaystyle\sum\limits_{j=1}^{k} \text{D}_{j} {R^{2}_{j}}}{\text{WD}} = \displaystyle\sum\limits_{j=1}^{k} \frac{\text{D}_{j}}{\text{WD}} {R^{2}_{j}}.  $$

According to Eq. 31, *R*^2^ can be seen as a weighted average of ${R^{2}_{1}},\ldots ,{R^{2}_{k}}$, with normalized weights D_1_/WD,…,D_*k*_/WD being the proportions of the within deviance due to each local deviance.

All the deviance measures introduced so far are summarized in Table 2, which provides the expressions, and a link between them, along with a short textual description.

**Table 2:** Proposed deviance measures, their description, and link between them

Deviance measure	Description
ED_*j*_	Local explained deviance in cluster *j*
RD_*j*_	Local residual deviance in cluster *j*
D_*j*_ = ED_*j*_ + RD_*j*_	Local (null) deviance in cluster *j*
$\text {EWD} = \displaystyle \sum\limits_{j=1}^k \text {ED}_j$	Explained within deviance
$\text {RWD} = \displaystyle \sum\limits_{j=1}^k \text {RD}_j$	Residual within deviance
$\text {WD} = \displaystyle \sum\limits_{j=1}^k \text {D}_j = \text {EWD} + \text {RWD}$	(Soft) within deviance
$\text {BD} = \displaystyle \sum\limits_{j=1}^k \text {BD}_j$	(Soft) between deviance
TD = BD + EWD + RWD	Null (or total) deviance
BD_*j*_	Soft contribution of cluster *j* to the between deviance.
NBD = BD/TD	Normalized between deviance
NEWD = EWD/TD	Normalized explained within deviance
NRWD = RWD/TD	Normalized residual within deviance
NED = 1 −NRWD	Normalized explained deviance
$R^2_j = \text {ED}_j/\text {D}_j$	Local deviance *R*^2^ in cluster *j*
$R^2 = \displaystyle \frac {\text {EWD}}{\text {WD}}= \displaystyle \sum\limits_{j=1}^k \frac {\text {D}_j}{\text {WD}} R^2_j$	Overall deviance *R*^2^

## Potential Limitations

Advancing the above ideas to define “adjusted” local and overall deviance *R*^2^ measures, similarly to the classical adjusted deviance *R*^2^ for GLMs (Guisan & Zimmermann, 2000, p. 167), to compare models with alternative nested/nonnested sets of covariates and/or with different number of latent groups, would seem natural. Below, we try to explain why, in our opinion, such an exercise makes no sense in the context of mixtures of GLMs.

### GLMs

Starting from Eq. 9, and similarly to the adjusted *R*^2^ for the OLS regression, the adjusted deviance *R*^2^ for GLMs is defined (Guisan & Zimmermann, 2000, p. 167) as

32 $$ \overline{R}^{2} = 1 - \frac{\frac{D\left( \widehat{\boldsymbol{\mu}},\boldsymbol{y};\widehat{\phi}\right)}{n-\left( d+1\right)}}{\frac{D\left( \bar{\boldsymbol{y}},\boldsymbol{y};\widehat{\phi}\right)}{n-1}}=1-\frac{n-1}{n-\left( d+1\right)}\left( 1-R^{2}\right),  $$

where $n-\left (d+1\right )$, and *n* − 1 represent the so-called number of degrees of freedom of $D\left (\widehat {\boldsymbol {\mu }},\boldsymbol {y};\widehat {\phi }\right )$ and $D\left (\bar {\boldsymbol {y}},\boldsymbol {y};\widehat {\phi }\right )$, respectively.

The primary attractiveness of $\overline {R}^{2}$ is that it imposes a penalty for adding additional independent variables to the GLM. The second related attractiveness of $\overline {R}^{2}$ is that it can be used to choose between nested/nonnested GLMs, with the aim of selecting the best set of explanatory variables (variable/model selection).

### Mixtures of GLMs

The arguments of Section 5.1 can be easily extended to the local and overall deviance *R*^2^ measures introduced in Section 4.3.

Adjusted local deviance *R*^2^.: In the spirit of Eq. 32, the adjusted local deviance *R*^2^ for mixtures of GLMs, in the generic *j* th group, could be defined as

33 $$ \overline{R}^{2}_{j}=1-\displaystyle\frac{\displaystyle\frac{\text{RD}_{j}}{\widehat{n}_{j}-\left( d+1\right)}}{\displaystyle\frac{\text{D}_{j}}{\widehat{n}_{j}-1}}.  $$

$\overline {R}^{2}_{j}$ implicitly assumes that the (soft) sample, of size $\widehat {n}_{j}$, is defined by the posterior probabilities $\widehat {z}_{ij}$, *i* = 1,…,*n*.

**Fig. 1:**
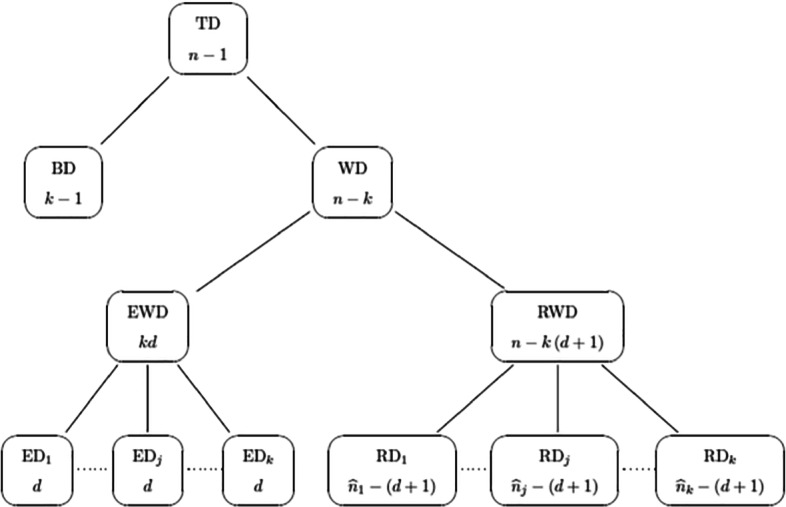
Hierarchical representation of the decomposition of the total deviance (TD). Each node of the hierarchy contains a particular deviance (on the top) along with its number of degrees of freedom (on the bottom)

**Fig. 2:**
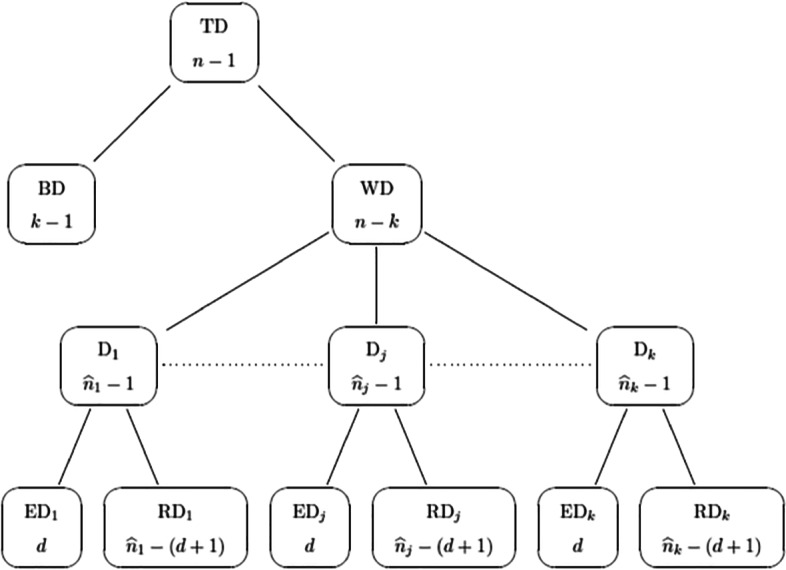
Hierarchical representation of the decomposition of the total deviance (TD). Each node of the hierarchy contains a particular deviance (on the top) along with its number of degrees of freedom (on the bottom)

According, for example, to Gujarati and Porter (2009), the number of degrees of freedom $\widehat {n}_{j}-\left (d+1\right )$ and $\widehat {n}_{j}-1$ in $\overline {R}^{2}_{j}$ is defined as the sample size minus the number of estimated parameters. Applying this rule, Figs. 1 and 2 show the number of degrees of freedom for all the deviances involved in our paper.

Under the assumption of fixed soft partition across all competing models, $ \overline {R}^{2}_{j}$ would share the attractive properties of the adjusted *R*^2^ in Eq. 32. Unfortunately, such an assumption is unrealistic. As said before, the mixture of GLMs needs to be re-estimated every time the set of explanatory variables and/or the number of groups change, and the estimated soft partition changes accordingly. Consequently, the comparison of $\overline {R}^{2}_{j}$-values from different models makes no sense.

Adjusted overall deviance *R*^2^.: By using the degrees of freedom of RWD and WD reported in Fig. 1, we can define the adjusted overall deviance *R*^2^ as

34 $$ \overline{R}^{2} = 1-\frac{\displaystyle\frac{\text{RWD}}{n-k\left( d+1\right)}}{\displaystyle\frac{\text{WD}}{n-k}}.  $$

Unfortunately, as for its local counterpart, $\overline {R}^{2}$ in Eq. 34 is not useful to choose between different mixtures of GLMs as the benchmark denominator WD in Eq. 34 changes every time a different model is fitted. This occurs because WD is only one of the components of TD (refer to Fig. 1). Whereby TD is the same for each fitted model, WD changes whenever the soft partition $\widehat {z}_{ij}$ does.

In summary, we can still use the local *R*^2^ in Eq. 33, and the overall *R*^2^ in Eq. 30, as descriptive measures of fit. Instead, we cannot use the adjusted local deviance *R*^2^ in Eq. 33, as well as the adjusted overall *R*^2^ in Eq. 34, in the variable/model selection step.

## Simulation Study

This simulation study has the goal of (i) investigating the behavior of the proposed local and overall deviance *R*^2^ measures in Eqs. 29 and 30, and (ii) assessing the role of the three terms in the decomposition of the deviance given in Eq. 26, under the exponential family distributions discussed in Section 3.3, namely, Gaussian, Poisson, and binomial distributions.

The simulation study considers the following set of conditions: (1) the class separation, under two levels: “small” and “large”; (2) the regression fit, under two levels: “poor” and “good”; and (3) the sample size *n*, under “small” size (100 units) and “large” size (1000 units). As for the class separation, the values “small” and “large” should not be considered in absolute terms, but simply conditioned to the regression fit factor. This results in a completely balanced design with 2^3^ = 8 crossed simulation conditions, which are summarized in Table 3.

**Table 3:** Simulation data conditions

	Class separation	Regression fit	Sample size (*n*)
Condition 1	“small”	“poor”	100
Condition 2	“large”	“poor”	100
Condition 3	“small”	“good”	100
Condition 4	“large”	“good”	100
Condition 5	“small”	“poor”	1000
Condition 6	“large”	“poor”	1000
Condition 7	“small”	“good”	1000
Condition 8	“large”	“good”	1000

For each data condition, we generated 250 data sets. To simplify the graphical representations, we take into account a single continuous covariate *X* (*d* = 1), generated from a standard normal distribution. The data generating process (DGP) is a mixture of *k* = 2 GLMs where the distribution of the response variable is assumed to be either Gaussian (Section 6.1), Poisson (Section 6.2) or binomial (Section 6.3). For the sake of space, we provide more insights about the first analysis, while we give brief comments for the other two examples. In the next subsections, we detail the parameters of the DGP and discuss the obtained simulation results for each DGP.

We conduct the whole analysis within the R environment (R Core Team, 2020). To fit mixtures of generalized linear regressions, we exploit the flexmix() function of the **flexmix** package (Leisch, 2004 and Grün & Leisch, 2008b). This function implements the EM algorithm to find ML estimates of the parameters. As the focus of the paper is not on computational aspects, we have decided to initialize the EM algorithm using the true partition of the generated data. Nonetheless, it might be of interest for the reader to understand how our measures perform in the real-life situation where cluster labels are unknown. This is why, in one of the three simulation studies—namely, the for the conditional Gaussian DGP—we decided to add a comparison of different initialization strategies for the EM algorithm.

Below we describe seven popular initialization strategies—TRUE.DGP, TRUE.clusters, RshortEM.1, RshortEM.10, PAM, *K*-means, and **mclust** (Scrucca et al., 2016)—which we use to obtain an initial (hard) partition.

TRUE.DGP.: The DGP is used to compute the posterior probabilities of cluster membership of the generated data (soft assignment). Then, the initial (hard) partition is obtained by means of the MAP criterion.
TRUE.clusters.: For simulated data the true cluster memberships are available, and can be used for initialization. This is our default strategy, the one used in all simulations.
Random short-EM.: This procedure, suggested by (Biernacki et al., 2003), consists in *S* short runs of the EM algorithm, each with *H* iterations, from different random positions. Each run of the EM algorithm is “short” because it is executed for a small number of iterations, without waiting for convergence. Then, the EM algorithm is run from the parameter vector providing the largest likelihood from these short runs of EM. We consider two values for the number of short runs ($S\in \left \{1,10\right \}$), while we fix *H* = 5. This gives rise to two alternatives that we name RshortEM.1 (when *S* = 1) and RshortEM.10 (when *S* = 10). To implement this initialization strategy, we use the initFlexmix() function of the **flexmix** package by specifying the arguments init = list("tol.em") and nrep = 1 (for RshortEM.1) and nrep = 10 (for RshortEM.10).
Partitional clustering.: Partitional clustering algorithms classify observations into *k* (specified by the user) clusters trying to minimize an objective function. A preliminary definition of “cluster center” is required. The most popular algorithms in this family are the following.
*k*-means.: The idea of *k*-means clustering ((Forgy, 1965) and (MacQueen, 1967)), which is the most commonly used partitional clustering algorithm, is to partition observations so that the within-cluster sum of squares is minimized; here, each cluster is represented by its center, corresponding to the mean of points assigned to that cluster. We carry out this EM-initialization strategy by performing a *k*-means cluster analysis 10 times (for stability sake), and subsequently using the partition from the solution with the lowest within-cluster sum of squares. For the purpose, we use the kmeans() function included in the **stats** package.
*k*-medoids (PAM).: In *K*-medoids clustering, each cluster is represented by one of the data points (called cluster medoid) such that the average dissimilarity between each cluster medoid and all the other members of its cluster is minimal. *k*-medoids clustering is a robust alternative to *k*-means clustering. The most common *k*-medoids clustering method is the partitioning around medoids algorithm (PAM;Kaufman & Rousseeuw, 1990). To implement the PAM-initialization strategy, we use the pam() function included in the **cluster** package (Maechler et al., 2019).

Both the methods are applied to the whole data $\left (X,Y\right )$.

Gaussian mixtures.: The use of Gaussian mixtures to obtain the initial partition is a further alternative. To fit these models, the EM algorithm is commonly used; it, in turn, requires an initialization strategy. To implement the EM algorithm to fit Gaussian mixtures, we refer to the Mclust() function of the **mclust** package. The latter allows fitting parsimonious variants of Gaussian mixtures. The EM algorithm is initialized according to partitions obtained from agglomerative hierarchical clustering procedures. In the analyses herein, we only consider the unconstrained Gaussian mixture, which is abbreviated as “VVV” in the package. Also in this case, we run the Mclust() function on the whole data $\left (X,Y\right )$.

This comparison is intended to raise the potential user’s awareness of how much the initialization stage can impact on the results.

### Gaussian case

**Fig. 3:**
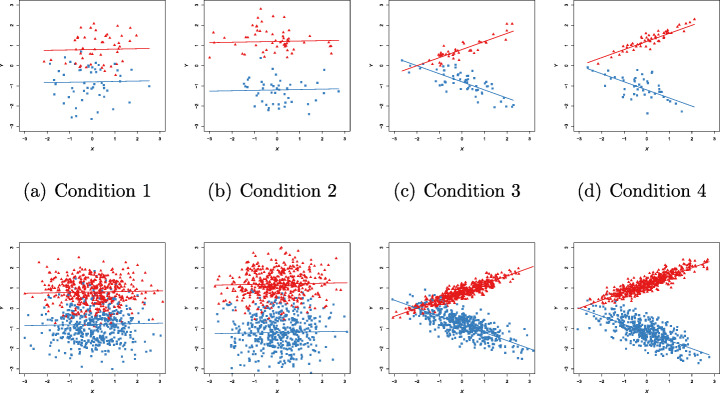
Gaussian case. Examples of generated data for each simulation condition in Table 3. Red triangles indicate first group membership, blue squares indicate second group membership

We report in Table 4 the parameters of the DGP for the Gaussian case, under each of the eight conditions in Table 3. The regression coefficients in clusters 1 and 2 are denoted as $\boldsymbol {\beta }_{1}=\left (\beta _{01},\beta _{11}\right )'$ and $\boldsymbol {\beta }_{2}=\left (\beta _{02},\beta _{12}\right )'$, respectively. Sample data sets, under each simulation condition, are provided in Fig. 3. The different colors refer to the underlying true classification (blue for cluster 1 and red for cluster 2).

**Table 4:** Gaussian case

Condition	1	2	3	4	5	6	7	8
*π*_1_	0.50	0.50	0.50	0.50	0.50	0.50	0.50	0.50
*β*_01_	− 0.80	− 1.20	− 0.80	− 1.20	− 0.80	− 1.20	− 0.80	− 1.20
*β*_11_	0.02	0.02	− 0.40	− 0.40	0.02	0.02	− 0.40	− 0.40
*β*_02_	0.80	1.20	0.80	1.20	0.80	1.20	0.80	1.20
*β*_12_	0.02	0.02	0.40	0.40	0.02	0.02	0.40	0.40
*σ*_1_	0.80	0.80	0.40	0.40	0.80	0.80	0.40	0.40
*σ*_2_	0.60	0.60	0.20	0.20	0.60	0.60	0.20	0.20

DGP-parameters for each simulation condition in Table 3

Table 5 shows, for each simulation condition, the Monte Carlo averages and standard deviations, over the 250 replications, of the following quantities: adjusted Rand index (ARI; (Hubert & Arabie, 1985)), to evaluate the agreement between the true partition and the MAP partition provided by the fitted model, ${R^{2}_{1}}$ (the deviance *R*^2^ in cluster 1), ${R^{2}_{2}}$ (the deviance *R*^2^ in cluster 2), *R*^2^ (the overall deviance *R*^2^), the normalized weights D_1_/WD (in cluster 1) and D_2_/WD (in cluster 2) defined in Eq. 31, and the NBD measure of cluster separation on the *y*-axis we introduced in Eq. 27.

**Table 5:** Gaussian case

Condition	1	2	3	4	5	6	7	8
ARI	0.414	0.772	0.834	0.969	0.542	0.831	0.842	0.973
	(0.175)	(0.119)	(0.075)	(0.033)	(0.046)	(0.025)	(0.024)	(0.011)
NBD	0.615	0.771	0.742	0.866	0.585	0.758	0.741	0.866
	(0.138)	(0.051)	(0.047)	(0.024)	(0.029)	(0.014)	(0.015)	(0.008)
${R^{2}_{1}}$	0.065	0.035	0.510	0.501	0.004	0.003	0.501	0.497
	(0.124)	(0.043)	(0.107)	(0.098)	(0.006)	(0.005)	(0.033)	(0.033)
${R^{2}_{2}}$	0.056	0.038	0.794	0.800	0.004	0.004	0.800	0.801
	(0.115)	(0.061)	(0.052)	(0.058)	(0.006)	(0.005)	(0.016)	(0.015)
D_1_/WD	0.473	0.499	0.299	0.292	0.507	0.499	0.285	0.285
	(0.155)	(0.076)	(0.081)	(0.083)	(0.065)	(0.021)	(0.026)	(0.024)
D_2_/WD	0.527	0.501	0.701	0.708	0.493	0.501	0.715	0.715
	(0.155)	(0.076)	(0.081)	(0.083)	(0.065)	(0.021)	(0.026)	(0.024)
*R*^2^	0.054	0.036	0.716	0.721	0.004	0.004	0.715	0.715
	(0.080)	(0.036)	(0.053)	(0.059)	(0.004)	(0.004)	(0.018)	(0.017)

Averages and standard deviations (in parentheses), over 250 Monte Carlo replicates, of different quantities

ARI and NBD can both be seen as cluster validation statistics. However, while the former works at an external level (using the external true partition as a benchmark), the latter works at an internal level (see, e.g., Kassambara, 2017, Chapter 13). The ARI values decrease as the overlap between clusters increases; this is what we expect because, for any model fitted to the data, it is more difficult to recover the true cluster memberships in the overlap region. In other words, the larger the overlap, the greater the difference between true and estimated partitions, the lower the ARI value. The NBD values decrease as the separation, once the points are projected along the *y*-axis, increases. So, in this case, the larger the “vertical” overlap, the lower the NBD value.

Concerning the evaluation of the average local and overall deviance *R*^2^s, we recall that the two clusters have an intercept of opposite sign (*β*_01_ = −*β*_02_) regardless of the simulation condition, the same slope (*β*_11_ = *β*_12_ = *β*_1_) for the simulation conditions 1, 2, 5, and 6, and slopes of opposite sign (*β*_11_ = −*β*_12_) for the remaining simulation conditions (see Table 4). However, the standard deviation in cluster 2 is always lower; this yields a generally larger local *R*^2^ in cluster 2 as the regression line fits better the data in this cluster. Moreover, when the absolute value of the slope is larger (refer to the simulation conditions 3, 4, 7, and 8 in Table 3), the local deviance *R*^2^s are larger too—showing the improvement of the local model with respect to the local intercept-only GLMs.

**Fig. 4:**
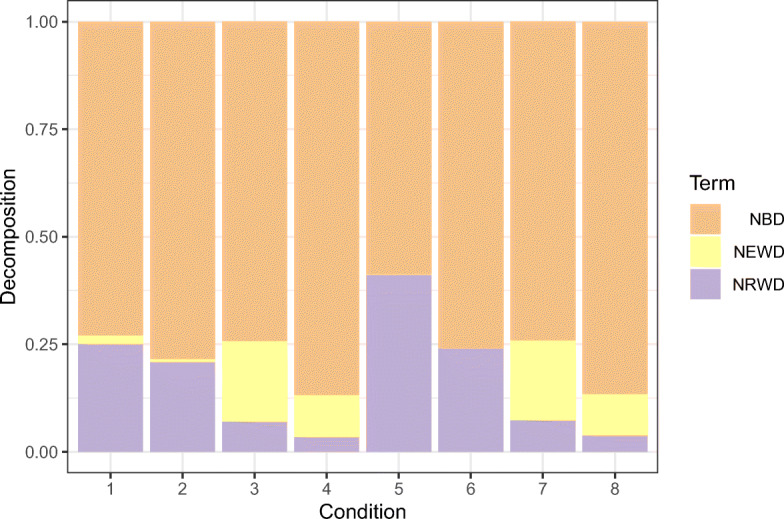
Gaussian case. Average terms, over 250 Monte Carlo replicates, of the total deviance decomposition in Eq. 26 for each of the simulation conditions in Table 3

The normalized weights depend on local deviances D_1_ and D_2_; so, the weight is large in the cluster where the difference between the saturated GLM and the intercept-only GLM is large too. The interpretation of the overall deviance *R*^2^ arises naturally—it is a simple weighted average of the local deviance *R*^2^s (${R^{2}_{1}}$ and ${R^{2}_{2}}$). Apart from the simulation conditions 1 and 5, we do not see any particular effect of the sample size on the obtained results. Finally, Fig. 4 gives a graphical representation of the normalized terms of the deviance decomposition in Eq. 4.1.

As said before, for this simulation case we also compare the results from the use of different initialization strategies. Tables 6–13 report the average values of ${R^{2}_{1}}$, ${R^{2}_{2}}$, *R*^2^, D_1_/WD, D_2_/WD, and NBD across the 250 replications for each of the considered simulation conditions in Table 3. Apart from the first two conditions, where the initialization strategy seems to play a role, there is an overall agreement between initialization strategies on all the considered indexes. As for the first two conditions, from Tables 6–7 we note that for a poor regression fit in each cluster and a small sample size, the initialization strategy has an impact on the determined classification and, as a by-product, on the results of our measures. Moreover, we also note how initializing the EM algorithm with the TRUE.DGP strategy produces worse results.

**Table 6:** Gaussian case, condition 1

	${R^{2}_{1}}$	${R^{2}_{2}}$	*R*^2^	D_1_/WD	D_2_/WD	NBD
TRUE.DGP	0.011	0.016	0.013	0.504	0.496	0.580
TRUE.clusters	0.065	0.056	0.054	0.473	0.527	0.615
*k*-means	0.072	0.055	0.056	0.461	0.539	0.611
PAM	0.074	0.053	0.057	0.459	0.541	0.610
mclust	0.085	0.094	0.085	0.478	0.522	0.581
EM.1	0.099	0.085	0.090	0.476	0.524	0.479
EM.10	0.108	0.085	0.093	0.472	0.528	0.487

Averages, over 250 Monte Carlo replicates, of different measures (by column) under various initialization strategies (by row)

**Table 7:** Gaussian case, condition 2

	${R^{2}_{1}}$	${R^{2}_{2}}$	*R*^2^	D_1_/WD	D_2_/WD	NBD
TRUE.DGP	0.013	0.018	0.016	0.500	0.500	0.758
TRUE.clusters	0.035	0.038	0.036	0.499	0.501	0.771
*k*-means	0.035	0.037	0.036	0.499	0.501	0.771
PAM	0.035	0.037	0.036	0.499	0.501	0.771
mclust	0.035	0.047	0.043	0.499	0.501	0.769
EM.1	0.084	0.066	0.082	0.501	0.499	0.574
EM.10	0.083	0.071	0.082	0.498	0.502	0.606

Averages, over 250 Monte Carlo replicates, of different measures (by column) under various initialization strategies (by row)

**Table 8:** Gaussian case, condition 3

	${R^{2}_{1}}$	${R^{2}_{2}}$	*R*^2^	D_1_/WD	D_2_/WD	NBD
TRUE.DGP	0.507	0.790	0.708	0.291	0.709	0.742
TRUE.clusters	0.510	0.794	0.716	0.299	0.701	0.742
*k*-means	0.510	0.794	0.716	0.299	0.701	0.742
PAM	0.510	0.794	0.716	0.299	0.701	0.742
mclust	0.510	0.794	0.716	0.299	0.701	0.742
EM.1	0.510	0.794	0.716	0.299	0.701	0.742
EM.10	0.510	0.794	0.716	0.299	0.701	0.742

Averages, over 250 Monte Carlo replicates, of different measures (by column) under various initialization strategies (by row)

**Table 9:** Gaussian case, condition 4

	${R^{2}_{1}}$	${R^{2}_{2}}$	*R*^2^	D_1_/WD	D_2_/WD	NBD
TRUE.DGP	0.504	0.796	0.710	0.298	0.702	0.866
TRUE.clusters	0.501	0.800	0.721	0.292	0.708	0.866
*k*-means	0.501	0.800	0.721	0.292	0.708	0.866
PAM	0.501	0.800	0.721	0.292	0.708	0.866
mclust	0.501	0.800	0.721	0.292	0.708	0.866
EM.1	0.499	0.797	0.718	0.293	0.707	0.863
EM.10	0.501	0.800	0.721	0.292	0.708	0.866

Averages, over 250 Monte Carlo replicates, of different measures (by column) under various initialization strategies (by row)

**Table 10:** Gaussian case, condition 5

	${R^{2}_{1}}$	${R^{2}_{2}}$	*R*^2^	D_1_/WD	D_2_/WD	NBD
TRUE.DGP	0.002	0.002	0.002	0.500	0.500	0.582
TRUE.clusters	0.004	0.004	0.004	0.507	0.493	0.585
*k*-means	0.004	0.004	0.004	0.496	0.504	0.586
PAM	0.004	0.004	0.004	0.497	0.503	0.586
mclust	0.004	0.004	0.004	0.504	0.496	0.583
EM.1	0.005	0.006	0.005	0.510	0.490	0.492
EM.10	0.005	0.007	0.006	0.512	0.488	0.568

Averages, over 250 Monte Carlo replicates, of different measures (by column) under various initialization strategies (by row)

**Table 11:** Gaussian case, condition 6

	${R^{2}_{1}}$	${R^{2}_{2}}$	*R*^2^	D_1_/WD	D_2_/WD	NBD
TRUE.DGP	0.002	0.003	0.003	0.500	0.500	0.757
TRUE.clusters	0.003	0.004	0.004	0.499	0.501	0.758
*k*-means	0.003	0.004	0.004	0.499	0.501	0.758
PAM	0.003	0.004	0.004	0.499	0.501	0.758
mclust	0.003	0.004	0.004	0.499	0.501	0.758
EM.1	0.006	0.006	0.006	0.498	0.502	0.354
EM.10	0.008	0.008	0.008	0.498	0.502	0.597

Averages, over 250 Monte Carlo replicates, of different measures (by column) under various initialization strategies (by row)

**Table 12:** Gaussian case, condition 7

	${R^{2}_{1}}$	${R^{2}_{2}}$	*R*^2^	D_1_/WD	D_2_/WD	NBD
TRUE.DGP	0.500	0.799	0.714	0.286	0.714	0.741
TRUE.clusters	0.501	0.800	0.715	0.285	0.715	0.741
k-means	0.501	0.800	0.715	0.285	0.715	0.741
PAM	0.501	0.800	0.715	0.285	0.715	0.741
mclust	0.501	0.800	0.715	0.285	0.715	0.741
EM.1	0.501	0.800	0.715	0.285	0.715	0.741
EM.10	0.501	0.800	0.715	0.285	0.715	0.741

Averages, over 250 Monte Carlo replicates, of different measures (by column) under various initialization strategies (by row)

**Table 13:** Gaussian case, condition 8

	${R^{2}_{1}}$	${R^{2}_{2}}$	*R*^2^	D_1_/WD	D_2_/WD	NBD
TRUE.DGP	0.498	0.800	0.713	0.287	0.713	0.866
TRUE.clusters	0.497	0.801	0.715	0.285	0.715	0.866
k-means	0.497	0.801	0.715	0.285	0.715	0.866
PAM	0.497	0.801	0.715	0.285	0.715	0.866
mclust	0.497	0.801	0.715	0.285	0.715	0.866
EM.1	0.491	0.791	0.706	0.288	0.712	0.856
EM.10	0.497	0.801	0.715	0.285	0.715	0.866

Averages, over 250 Monte Carlo replicates, of different measures (by column) under various initialization strategies (by row)

### Poisson case

We report in Table 14 the parameters of the DGP for the Poisson case, under each simulation condition in Table 3.

**Table 14:** Poisson case

Condition	1	2	3	4	5	6	7	8
*π*_1_	0.50	0.50	0.50	0.50	0.50	0.50	0.50	0.50
*β*_01_	− 0.75	− 1.00	− 0.75	− 1.00	− 0.75	− 1.00	− 0.75	− 1.00
*β*_11_	0.15	0.15	1.50	1.50	0.15	0.15	1.50	1.50
*β*_02_	1.50	2.00	1.50	2.00	1.50	2.00	1.50	2.00
*β*_12_	− 0.07	− 0.07	0.50	0.50	− 0.07	− 0.07	0.50	0.50

DGP-parameters for each simulation condition in Table 3

**Fig. 5:**
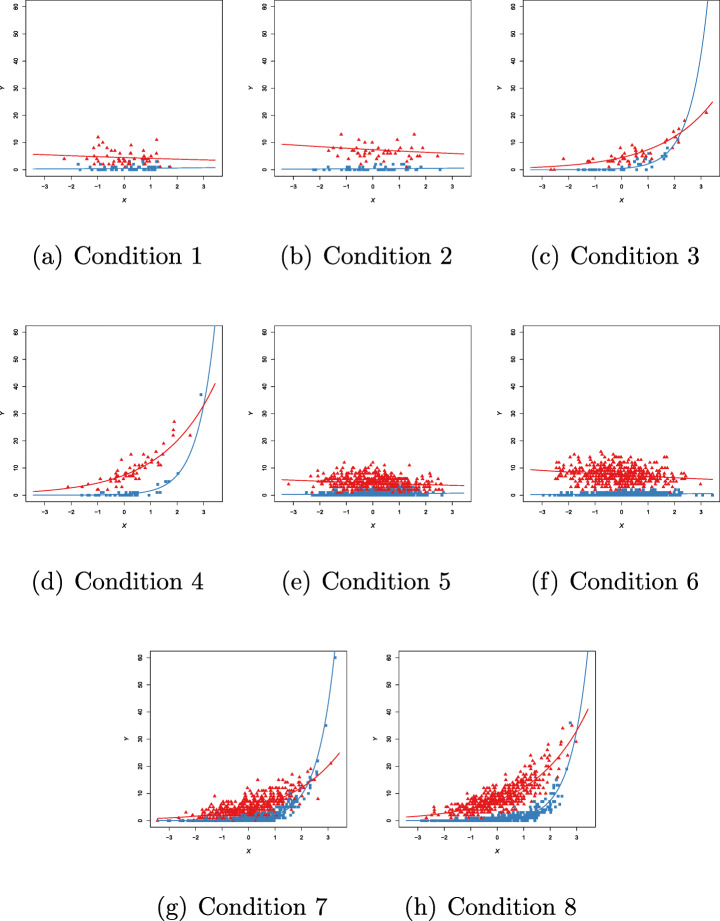
Poisson case. Examples of generated data for each simulation condition in Table 3. Red triangles indicate first group membership, and blue squares indicate second group membership

We give examples of generated data in Fig. 5. Also in this case, we use blue for cluster 1 and red for cluster 2.

Table 15 shows the obtained results for each simulation condition. Under the odd simulation conditions, clusters are poorly separated, and this affects cluster recovery—showing relatively small ARI values. Under the simulation conditions 3, 4, 7, and 8, the NBD values are small because, once projected on the *y*-axis, the clusters overlap in such a way that the fitted means $\bar {y}_{1}$ and $\bar {y}_{2}$ are close and, consequently, the difference between the intercept-only model and the local intercept-only model is small (see Fig. 5). The local deviance *R*^2^s are small when the local slope is close to zero (simulation conditions 1, 2, 5, and 6). This occurs because the fitted and local intercept-only models are somewhat similar.

**Table 15:** Poisson case

Condition	1	2	3	4	5	6	7	8
ARI	0.699	0.931	0.612	0.895	0.722	0.942	0.644	0.911
	(0.104)	(0.053)	(0.118)	(0.061)	(0.033)	(0.015)	(0.030)	(0.019)
NBD	0.636	0.796	0.262	0.481	0.642	0.798	0.248	0.493
	(0.053)	(0.026)	(0.114)	(0.107)	(0.014)	(0.009)	(0.049)	(0.042)
${R^{2}_{1}}$	0.064	0.049	0.760	0.735	0.016	0.011	0.786	0.748
	(0.093)	(0.074)	(0.120)	(0.135)	(0.014)	(0.011)	(0.045)	(0.045)
${R^{2}_{2}}$	0.045	0.053	0.528	0.652	0.023	0.036	0.546	0.665
	(0.057)	(0.058)	(0.115)	(0.081)	(0.014)	(0.017)	(0.035)	(0.026)
D_1_/WD	0.482	0.475	0.615	0.519	0.484	0.465	0.637	0.516
	(0.092)	(0.072)	(0.126)	(0.115)	(0.028)	(0.022)	(0.055)	(0.051)
D_2_/WD	0.518	0.525	0.385	0.481	0.516	0.535	0.363	0.484
	(0.092)	(0.072)	(0.126)	(0.115)	(0.028)	(0.022)	(0.055)	(0.051)
*R*^2^	0.056	0.053	0.684	0.706	0.020	0.024	0.702	0.710
	(0.059)	(0.050)	(0.097)	(0.089)	(0.009)	(0.010)	(0.044)	(0.031)

Averages and standard deviations (in parentheses), over 250 Monte Carlo replicates, of different quantities

**Fig. 6:**
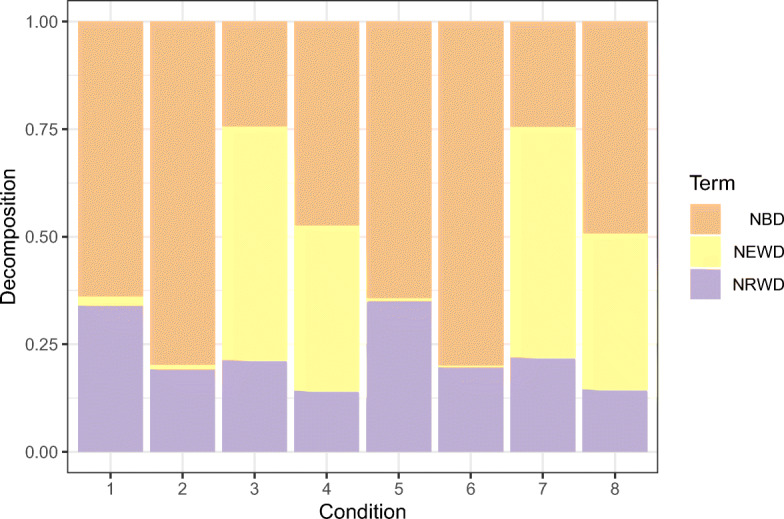
Poisson case. Average terms, over 250 Monte Carlo replicates, of the total deviance decomposition in Eq. 26 for each of the simulation conditions in Table 3

Figure 6 gives a graphical representation of the normalized terms of the deviance decomposition in Eq. 4.1. Also in this case, we do not see any particular effect of the sample size on the obtained results.

### Binomial Case

Table 16 reports the parameters of the DGP for the binomial case, under each simulation condition in Table 3; the number of trials has been fixed to *m* = 10.

**Table 16:** Binomial case (*m* = 10)

Condition	1	2	3	4	5	6	7	8
*π*_1_	0.50	0.50	0.50	0.50	0.50	0.50	0.50	0.50
*β*_01_	− 1.00	− 2.00	− 1.00	− 2.00	− 1.00	− 2.00	− 1.00	− 2.00
*β*_11_	0.01	0.01	2.00	2.00	0.01	0.01	2.00	2.00
*β*_02_	1.00	2.00	1.00	2.00	1.00	2.00	1.00	2.00
*β*_12_	0.01	0.01	2.00	2.00	0.01	0.01	2.00	2.00

DGP-parameters for each simulation condition in Table 3

**Fig. 7:**
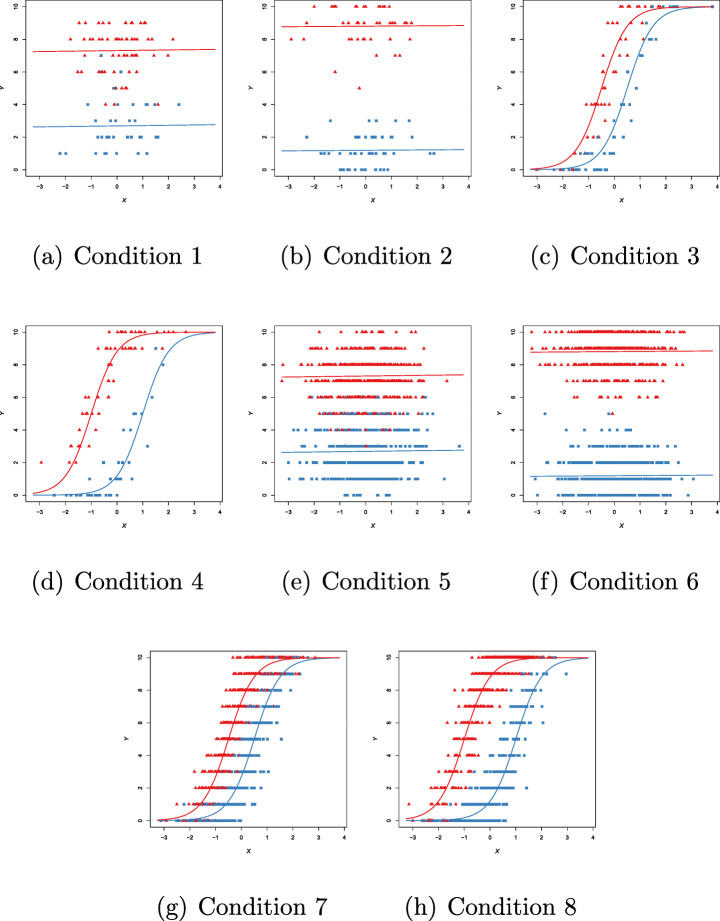
Binomial case (*m* = 10). Examples of generated data for each simulation condition in Table 3. Red triangles indicate first group membership, blue squares indicate second group membership

In Fig. 7, we plot 8 sample data sets for each of the 8 simulation conditions in Table 3.

Table 17 shows the obtained results for each simulation condition. In this example, it is interesting to note the small values of the NBD term under the simulation conditions 3, 4, 7, and 8 (refer to Figs. 7(c), (d), (g), and (h)); in these cases, the clusters overlap a lot when the points are projected on the *y*-axis and this impacts on the NBD values. Moreover, the local slopes are the same in both clusters (*β*_11_ = *β*_12_ = *β*_1_) regardless of the simulation condition: local and overall deviance *R*^2^s are small when *β*_1_ = 0.01, and large when *β*_1_ = 2.

**Table 17:** Binomial case (*m* = 10)

Condition	1	2	3	4	5	6	7	8
ARI	0.757	0.992	0.499	0.885	0.755	0.992	0.533	0.889
	(0.088)	(0.018)	(0.116)	(0.068)	(0.025)	(0.006)	(0.033)	(0.020)
NBD	0.667	0.848	0.137	0.417	0.665	0.848	0.141	0.413
	(0.040)	(0.017)	(0.039)	(0.065)	(0.012)	(0.005)	(0.012)	(0.022)
${R^{2}_{1}}$	0.028	0.019	0.812	0.785	0.003	0.002	0.808	0.787
	(0.037)	(0.026)	(0.037)	(0.051)	(0.004)	(0.003)	(0.010)	(0.016)
${R^{2}_{2}}$	0.033	0.020	0.810	0.786	0.003	0.001	0.806	0.785
	(0.040)	(0.027)	(0.039)	(0.049)	(0.005)	(0.002)	(0.011)	(0.017)
D_1_/WD	0.496	0.506	0.502	0.500	0.498	0.501	0.503	0.503
	(0.073)	(0.073)	(0.090)	(0.077)	(0.020)	(0.024)	(0.026)	(0.024)
D_2_/WD	0.504	0.494	0.498	0.500	0.502	0.499	0.497	0.497
	(0.073)	(0.073)	(0.090)	(0.077)	(0.020)	(0.024)	(0.026)	(0.024)
*R*^2^	0.031	0.019	0.813	0.788	0.003	0.002	0.807	0.786
	(0.030)	(0.019)	(0.028)	(0.036)	(0.003)	(0.002)	(0.008)	(0.012)

Averages and standard deviations (in parentheses), over 250 Monte Carlo replicates, of different quantities

**Fig. 8:**
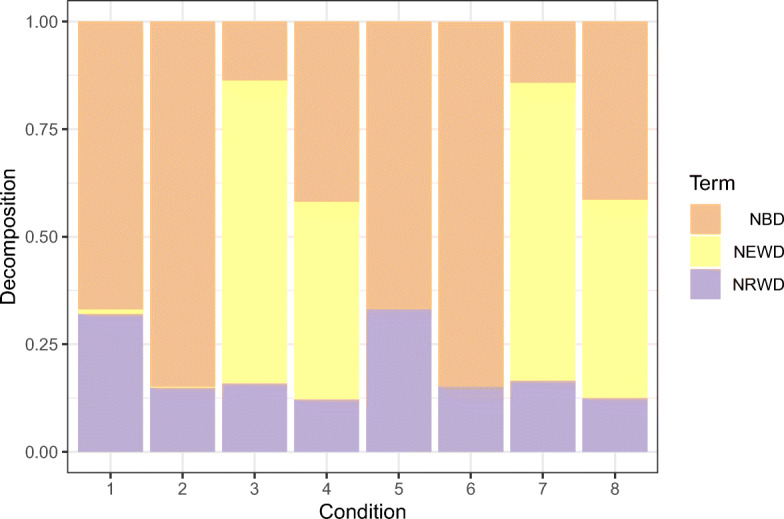
Binomial case (*m* = 10). Average terms, over 250 Monte Carlo replicates, of the total deviance decomposition in Eq. 26 for each of the simulation conditions in Table 3

Figure 8 gives a graphical representation of the normalized terms of the deviance decomposition in Eq. 4.1. Also in this case, we do not observe any particular effect of the sample size on the obtained results.

## Clusters of COVID-19 Cases in Italy Before and After Social Restrictions

The Coronavirus disease 2019—better known as COVID-19—is caused by the SARS-CoV-2 virus that has appeared in Wuhan (China) in late 2019. Quickly, the virus has spread within and outside China to the entire world. Italy has had 12462 confirmed cases according to the Istituto Superiore di Sanità as of March 11, 2020, and 827 deaths. Only China has recorded more deaths due to this COVID-19 outbreak. Why the spread has been so fast is a major concern. The average age of those who died in Italy was 81 years. More than two-thirds of these patients had risk factors like diabetes, cardiovascular diseases, or cancer, or were former smokers (Remuzzi & Remuzzi, 2020). In addition to risk factor prevalences, the peculiar shape of the Italian territory—very few uninhabited and isolated lands—may have facilitated the COVID-19’s pattern of spread.

In this application, we analyze data from the Italian Civil Protection Department (“Dipartimento di Prodezione Civile” in Italian, DPC hereafter) that are publicly available^1^. The DPC website contains several daily time-series regarding the COVID-19 outbreak in Italy, at different levels of aggregation, starting from 2020, February 24. We focus on the county-level records, the least aggregated data available.

The aim of this real–data analysis is to find geographic clusters of COVID-19 prevalence, before and after the introduction of the two most severe social restrictions, respectively enforced in 2020, March 11 (lock-down of all commercial activities and retail sales, except for grocery and staple food stores, and pharmacies), and March 20 (lock-down of city parks) by the Italian Government. To do so, we specify a conditional Poisson mixture, in which the number of positives is regressed on each county’s geographic coordinates—offsetting for the total population of each county. The analysis will be carried out separately for the two time points.

On the March 11 data set, we select the number of components *k* ranging from 1 to 4. We choose not to go beyond 4 clusters to avoid overfitting solutions, due to the relatively small sample size (*n* = 106 counties), and to guarantee class interpretability. For each value of *k*, we implement the Random short-EM initialization strategy. To guarantee a stable solution, we decided to set the number of replicates to *S* = 100.

BIC values are 18379.886, 3843.310, 2305.327, and 1371.599, for *k* = 1,2,3,4, respectively. For model-based clustering with count data, entropy-based criteria for selecting the mixture order may be used as well. In our real–data example, ICL displays a similar trend to BIC—3845.215, 2309.169, and 1394.763, respectively for *k* = 2,3,4. To illustrate our proposal, we will present local, and overall fit measures for all 4 models. For further analyses—regression tables, clustering results and comparison with March 26 data—we will focus on the minimum BIC solution. To avoid label switching and to enhance class interpretation, the estimated posteriors from March 11 data will be used to initialize flexmix() to fit March 26 data.

Table 18 presents class proportions, local, and overall fit measures for March 11 data. We observe an overall *R*^2^ around 0.45 for the 1-class solution, and 0.82 for *k* = 2. In the 2-class solution, the two classes have very uneven sample sizes (0.169 and 0.831), with the smaller group having a better regression fit than the other one—taking values 0.864 and 0.768, respectively—and also a larger contribution to the overall within deviance.

**Table 18:** Local and overall fit measures for March 11, 2020, Italian county-level data

Cluster	1	2	3	4
	*k* = 1			
*π*_*j*_	1.000	−	−	−
${R^{2}_{j}}$	0.448	−	−	−
D_*j*_/WD	1.000	−	−	−
*R*^2^	0.448			
NBD	0.000			
	*k* = 2			
*π*_*j*_	0.169	0.831	−	−
${R^{2}_{j}}$	0.864	0.768	−	−
D_*j*_/WD	0.521	0.479	−	−
*R*^2^	0.818			
NBD	0.458			
	*k* = 3			
*π*_*j*_	0.130	0.680	0.190	−
${R^{2}_{j}}$	0.909	0.875	0.860	−
D_*j*_/WD	0.511	0.164	0.326	−
*R*^2^	0.888			
NBD	0.576			
	*k* = 4			
*π*_*j*_	0.520	0.092	0.126	0.262
${R^{2}_{j}}$	0.913	0.985	0.978	0.972
D_*j*_/WD	0.090	0.250	0.421	0.239
*R*^2^	0.972			
NBD	0.366			

A similar pattern emerges for *k* = 3: two small classes with proportions 0.130 and 0.190, and a larger one with proportion 0.680. The two smaller classes deliver a higher local *R*^2^, and a greater contribution to the overall within deviance than the largest one. Similarly, also the NBD increases.

In the case with four classes (*k* = 4), there are two large groups of relative sizes 0.520 and 0.262, and two smaller groups with proportions 0.092 and 0.126. Interestingly, we observe that the two smaller groups have a relatively greater local *R*^2^ than the larger groups. Group 3 (with $\hat {\pi }_{3}=0.126$) has the most relevant contribution to the overall within deviance. Note also that NBD, with respect to the *k* = 3 case, reduces to 0.366 due to a greater overlap among the groups.

**Fig. 9:**
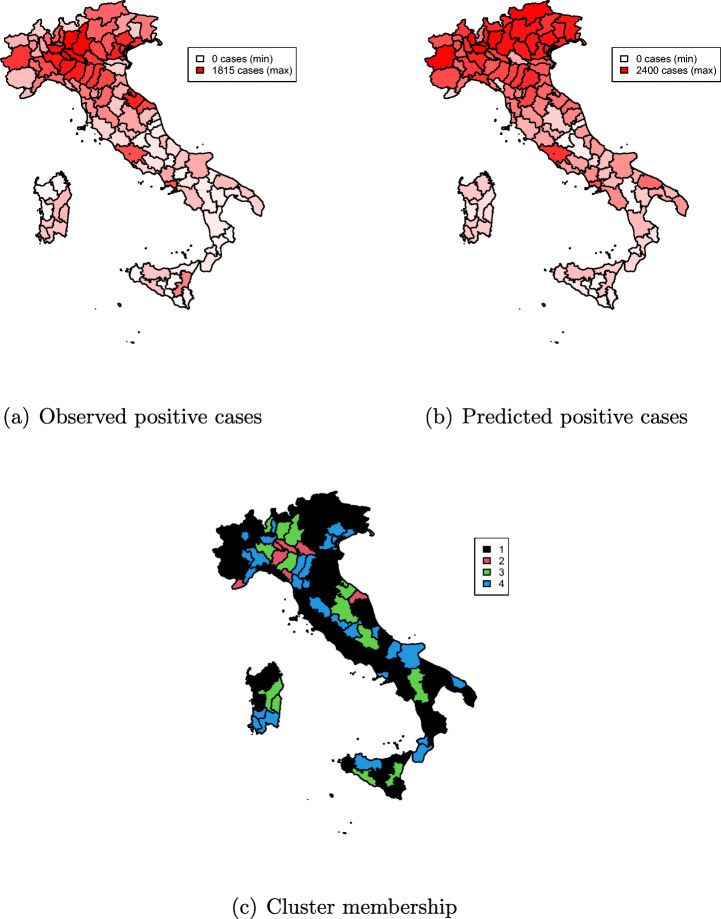
Cluster membership, predicted, and observed number of COVID-19-positive cases as of March 11, 2020

Table 19 reports regression results for March 11 data. Both intercepts and regression coefficients differ significantly across group. The first (largest) group has the smallest (statistically significant) latitude coefficient, and a non-significant coefficient for longitude. Counties belonging to this group are spread all over Italy and have a relatively smaller number of positives as of March 11 (Fig. 9) compared to counties belonging to the other groups.

**Table 19:** Cluster-specific regression parameters for March 11, 2020, Italian county-level data with *k* = 4

Cluster	1	2	3	4
*π*_*j*_	0.520	0.092	0.126	0.262
intercept	− 25.837^∗∗∗^	− 67.978^∗∗∗^	− 32.031^∗∗∗^	− 27.068^∗∗∗^
	(0.912)	(2.132)	(1.180)	(0.847)
latitude	0.364^∗∗∗^	1.403^∗∗∗^	0.528^∗∗∗^	0.424^∗∗∗^
	(0.019)	(0.043)	(0.022)	(0.017)
longitude	− 0.001	− 0.115^∗∗∗^	0.130^∗∗∗^	− 0.037^∗∗^
	(0.014)	(0.030)	(0.020)	(0.012)
${R^{2}_{j}}$	0.913	0.985	0.978	0.972
*R*^2^	0.972			
NBD	0.366			

Standard errors in parentheses. Significance codes: *p*-value ≈ 0 ‘^∗∗∗^’, ≤ 0.001 ‘^∗∗^’, ≤ 0.01 ‘^∗^’, ≤ 0.05 ‘^⋅^’

The second class is the one with the smallest size, and has a relatively large and positive coefficient on latitude, and a negative one for longitude: counties belonging to this group are located in the north-western part of Italy and on average have a relatively larger number of positives as of March 11.

Group 3 is the second largest in terms of average number of positives—some of the northern counties where the pandemic struck the hardest in the first place. Note that some counties in the center, and south of the country are classified to this group as well, although with a smaller number of absolute positives than northern counties from the same group. This is due to the fact that the number of cases per inhabitant are anyway comparable, and the model accounts for this through the offset term.

The fourth group has a similar effect of latitude on the response as in group 3, but a small and negative coefficient for longitude. We observe from the map (Fig. 9(c)) that groups 3 and 4 are constituted by counties located along highways. Yet, group 4 has an average smaller COVID-19 prevalence than group 3. Overall, the model seems to fit the data well in terms of observed (Fig. 9(a)) against predicted (Fig. 9(b)) number of positives. Interestingly, the prevalence of COVID-19 in northern counties is smoothed upward.

Table 20 displays local, overall fit measures, and regression results for March 26 data. The overall pattern is similar to March 22 data, still with noteworthy differences. The biggest class has a relatively lower size, with both local *R*^2^ and D_*j*_/WD indicating a better local fit, and a larger contribution to the overall within variation—the class is now more homogeneous. By contrast, class two has grown in size, though with a relatively lower average number of positives (Fig. 9(c)), and a poorer local regression fit.

**Table 20:** Cluster-specific regression parameters for March 26, 2020, Italian county-level data with *k* = 4, along with local and overall fit measures

Cluster	1	2	3	4
*π*_*j*_	0.427	0.140	0.174	0.259
intercept	− 18.998^∗∗∗^	− 26.981^∗∗∗^	− 30.804^∗∗∗^	− 17.392^∗∗∗^
	(0.197)	(0.787)	(0.383)	(0.197)
latitude	0.284^∗∗∗^	0.524^∗∗∗^	0.510^∗∗∗^	0.262^∗∗∗^
	(0.004)	(0.014)	(0.007)	(0.003)
longitude	− 0.066^∗∗∗^	− 0.137^∗∗∗^	0.238^∗∗∗^	− 0.070^∗∗^
	(0.003)	(0.013)	(0.008)	(0.007)
${R^{2}_{j}}$	0.941	0.832	0.969	0.976
D_*j*_/WD	0.185	0.113	0.423	0.279
*R*^2^			0.950	
NBD			0.117	

Standard errors in parentheses. Significance codes: *p*-value ≈ 0 ‘^∗∗∗^’, ≤ 0.001 ‘^∗∗^’, ≤ 0.01 ‘^∗^’, ≤ 0.05 ‘^⋅^’

**Fig. 10:**
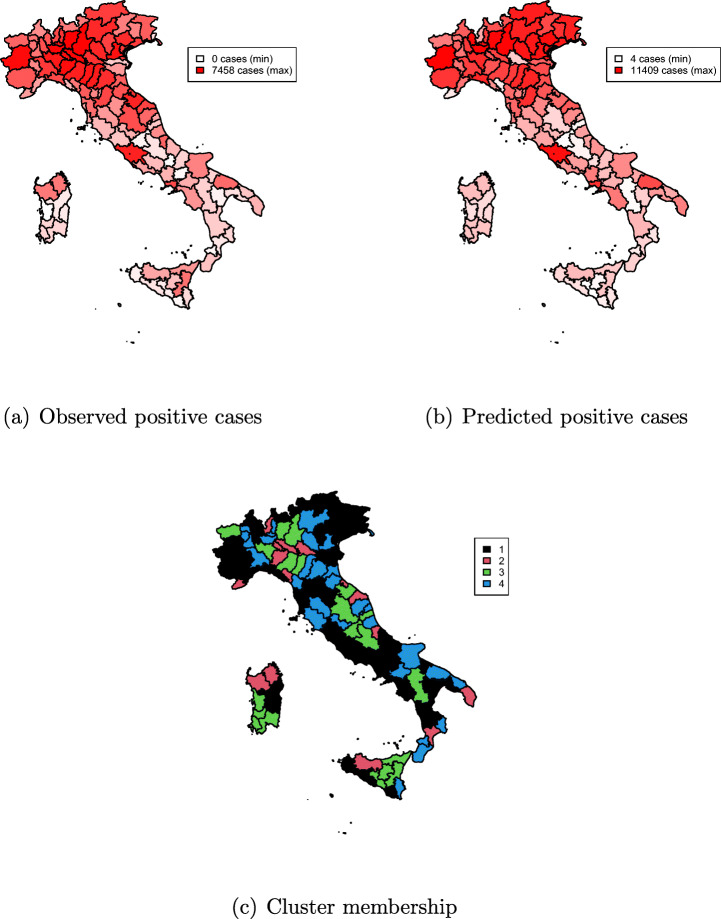
Cluster membership, predicted, and observed number of COVID-19-positive cases as of March 26, 2020

On average, counties with largest number of positives relative to inhabitants belong to group 3. Group 4 is the second smallest average number of positives. Interestingly, from the regression output (Table 20) we observe that coefficients for latitude are all smaller than for March 11 data, indicating that the pandemic has spread all over the country (see also Fig. 10(a), (b), and (c)). All in all, it seems that the spread of the virus is relatively more even as of March 26, compared to March 11.

## Conclusions

In this paper, we have proposed several deviance-based measures to evaluate the goodness/lack of fit for mixtures of GLMs, at both cluster and whole-sample level; this approach extends usual indices for a single GLM. Our aim was to gain further insights about the fitted model focusing on the behavior within and between clusters.

The proposals have been illustrated by means of a large simulation study that covered Gaussian, Poisson, and binomial responses as special cases of the exponential family distributions, and an application on COVID-19 Italian data. In the COVID-19 data application, we observed that, before and after the two social restriction measures enforced by the Italian government, the spread of the pandemic has become more evenly distributed along the country.

At a more general level, the proposed fit measures can be very useful in applied research with clusterwise regression analysis to assess model fit, as well as to validate the clustering solution(s). Regarding software implementation, a commented R script computing all goodness of fit quantities can be found as an online supplement to this manuscript.

It is important to remark that the use of these measures to perform model selection, namely, to compare models with a different nested/nonnested sets of covariates and/or a different number of latent groups, is completely deceptive. The reason being that different models deliver different soft groupings, and there is no direct link among groupings from different models. Therefore, even if we were able to adjust our measures for the degrees of freedom of each competing model, the resulting comparison would intrinsically not make sense. Such an issue was already noted by (Ingrassia & Punzo, 2020) in the case of *R*^2^ measures for mixtures of linear models.

Although these deviance-based measures have been introduced exploiting the usual EM algorithm, their validity goes beyond it. Our measures also work when the parameters of the model are estimated by variants of the EM algorithm, such as the stochastic EM (SEM; Diebolt & Ip, 1996) or the classification EM (CEM; Celeux & Govaert, 1992). Such algorithms are well-known in the mixture modeling literature — for instance, their implementation is also available in the **flexmix** package (Grün & Leisch, 2008b).

For both variants, an additional step is added between the E- and M-steps, where the estimated a posteriori probabilities are used to assign each observation to only one component. For the SEM algorithm this assignment is determined in a stochastic way by randomly drawing memberships for each unit *i* from a multinomial distribution with probabilities $\widehat {z}_{i1},\ldots ,\widehat {z}_{ik}$, *i* = 1,…,*n*. By contrast, the assignment is deterministic for the CEM algorithm. Therefore, to extend our fit measures to these cases, we simply need to replace the soft cluster memberships defined in Section 3.2 with hard (crisp) 0/1 memberships.

Future work can focus on the extension of the results of this paper to mixtures of generalized nonlinear models, the family of models recently introduced by Omerovic (2019) in her Ph.D. thesis.

## Data Availability

The real data set on COVID spread analyzed during the current study is public and can be downloaded from https://opendatamds.maps.arcgis.com/apps/dashboards/0f1c9a02467b45a7b4ca12d8ba296596.

## Footnotes

1 https://opendatadpc.maps.arcgis.com/apps/opsdashboard/index.html#/b0c68bce2cce478eaac82fe38d4138b1
